# Cystatin A suppresses tumor cell growth through inhibiting epithelial to mesenchymal transition in human lung cancer

**DOI:** 10.18632/oncotarget.23505

**Published:** 2017-12-20

**Authors:** Yunxia Ma, Yuan Chen, Yong Li, Katja Grün, Alexander Berndt, Zhongwei Zhou, Iver Petersen

**Affiliations:** ^1^ Institute of Pathology, University Hospital Jena, Friedrich Schiller University Jena, Jena, Germany; ^2^ Department of Internal Medicine I, University Hospital Jena, Jena, Germany; ^3^ Leibniz Institute for Age Research, Fritz Lipmann Institute, Jena, Germany; ^4^ Current/Present address: Institute of Pathology, SRH Wald-Klinikum Gera, Gera, Germany

**Keywords:** cystatin A, tumor suppressor, epithelial to mesenchymal transition, DNA methylation, lung cancer

## Abstract

Cystatin A (*CSTA*), belonging to type 1 cystatin super-family, is expressed primarily in epithelial and lymphoid tissues for protecting cells from proteolysis of cytoplasmic and cytoskeletal proteins by cathepsins B, H and L. CSTA acts as a tumor suppressor in esophageal cancer, however, its role in lung cancer has not yet been elucidated. Here we found that CSTA was down-regulated in all lung cancer cell lines compared to normal lung epithelial cells. *CSTA* was restored in most lung cancer cell lines after treatment with demethylation agent 5-aza-2-deoxycytidine and deacetylation agent Trichostatin. Bisulfite sequencing revealed that *CSTA* was partially methylated in the promoter and exon 1. In primary lung tumors, squamous cell carcinoma (SCC) significantly expressed more CSTA compared to adenocarcinoma (p<0.00001), and higher expression of CSTA was significantly associated with lower tumor grade (p<0.01). CSTA stable transfection reduced the activity of cathepsin B and inhibited the ability of colony formation, migration and invasion, and enhanced gemcitabine-induced apoptosis. CSTA overexpression resulted in reduced activity of ERK, p-38, and AKT. Additionally, CSTA overexpression led to a mesenchymal to epithelial transition (MET) and prevented the TGF-β1-induced epithelial to mesenchymal transition (EMT) through inhibiting the ERK/MAPK pathway. In conclusion, our date indicate 1) epigenetic regulation is associated with *CSTA* gene silencing; 2) CSTA exerts tumor suppressive function through inhibiting MAPK and AKT pathways; 3) Overexpression of CSTA leads to MET and prevents TGF-β1-induced EMT by modulating the MAPK pathway; 4) CSTA may be a potential biomarker for lung SCC and tumor differentiation.

## INTRODUCTION

Lung cancer is the first leading cause of cancer-related death in the United States with 222,500 new cases being estimated to occur in 2017 [1]. Through the development of advanced technologies and methods in lung cancer treatment, the 5-year survival rate was just slightly increased from 13% to 18% in the United States [2]. Thus, a better understanding of biological signaling involved in tumor promotion and suppression is required to identify valuable diagnostic/prognostic biomarkers and to develop novel therapeutic strategies.

A cancer therapy strategy based on cystatin functions was proposed in recent years [3]. Cystatins, considered cysteine protease inhibitors, have emerged as important regulators in a multitude of physiological and pathophysiological processes involved in cell survival, proliferation, differentiation, cell signaling and immunomodulation [3, 4]. Cystatin A (CSTA, Stefin A), one of the type 1 cystatin super-family members, is mainly intracellularly located and it can also be found in body fluids [5]. CSTA is expressed primarily in epithelial and lymphoid tissues for protecting cells from proteolysis of cytoplasmic and cytoskeletal proteins by inhibiting cathepsin B, H and L [4, 5]. Mutations in CSTA are associated with skin fragility phenotype including exfoliative ichthyosis and acral peeling skin syndrome [6, 7].

Previously, by using suppression subtractive hybridization (SSH), we observed that *CSTA* mRNA was down-regulated in lung cancer cell lines (D51, H226 and H2170) compared to normal human bronchial epithelial cells (HBEC) [8]. Later, Bianchi *et al*. confirmed that CSTA expression was markedly decreased in advanced lung tumors compared with early ones [9]. CSTA was found to be down-regulated in esophageal squamous cell cancer, prostate cancer, skin cancer, and breast cancer [10–13]. In lung cancer and head and neck cancer, positive staining of CSTA was significantly related to squamous carcinoma (SCC) compared to adenocarcinoma (ADC) [14, 15], additionally, in head and neck cancer, reduced CSTA expression was correlated with tumor recurrence [15]. However, so far the function of CSTA has not yet been elucidated in lung cancer.

Epithelial-to-mesenchymal transition (EMT) plays a critical role in tumor progression and malignant transformation. Cystatin superfamily was proposed to be able to inhibit EMT through preventing the cathepsin-induced proteolytic cleavage of extracellular matrix (ECM) components, adhesion proteins and adherens junctions [16]. Cystatin C suppresses TGF-β induced EMT by preventing cytoskeletal rearrangements and E-cadherin in breast cancer cells [17]. Cystatin D inhibits EMT through down-regulation of EMT transcription factors in colon cancer [18]. **Additionally, Snake** venom cystatin prevents tumor cell invasion and metastasis through reduction of proteinase activity and EMT in hepatocellular carcinoma cells [19]. Loss of CSTA leads to decreased cell-cell adhesion by disrupting desmosomal structures in epidermoid carcinoma cells [20]. However, there is limited knowledge concerning the role of CSTA on cell phenotype transition during lung cancer development.

Therefore, the study was aimed to analyze the epigenetic regulation and function of CSTA with special focus on EMT in lung cancer cells. The clinical relevance of CSTA was also evaluated in primary lung tumors.

## RESULTS

### CSTA is down-regulated in lung cancer

**Figure 1:**
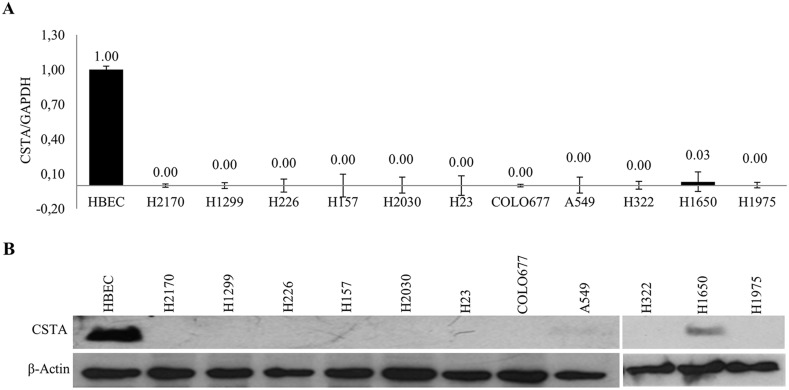
Expression of CSTA in lung cancer cell lines **(A)** mRNA expression of *CSTA* in 11 lung cancer cell lines and normal cells HBEC was analyzed by real-time RT-PCR. Relative mRNA expression is presented in comparison with reference HBEC expression. The data are presented as the means of three independent experiments ± standard deviation. **(B)** CSTA protein expression was determined by WB. β-actin was used as a loading control.

Northern blot (NB), real-time RT-PCR and western blot (WB) showed that CSTA was not detected in all lung cancer cell lines at both mRNA (Figure 1A and Supplementary Figure 1A) and protein (Figure 1B) levels except H1650 compared to HBEC and Small airway epithelial cells (SAEC).

**Figure 2:**
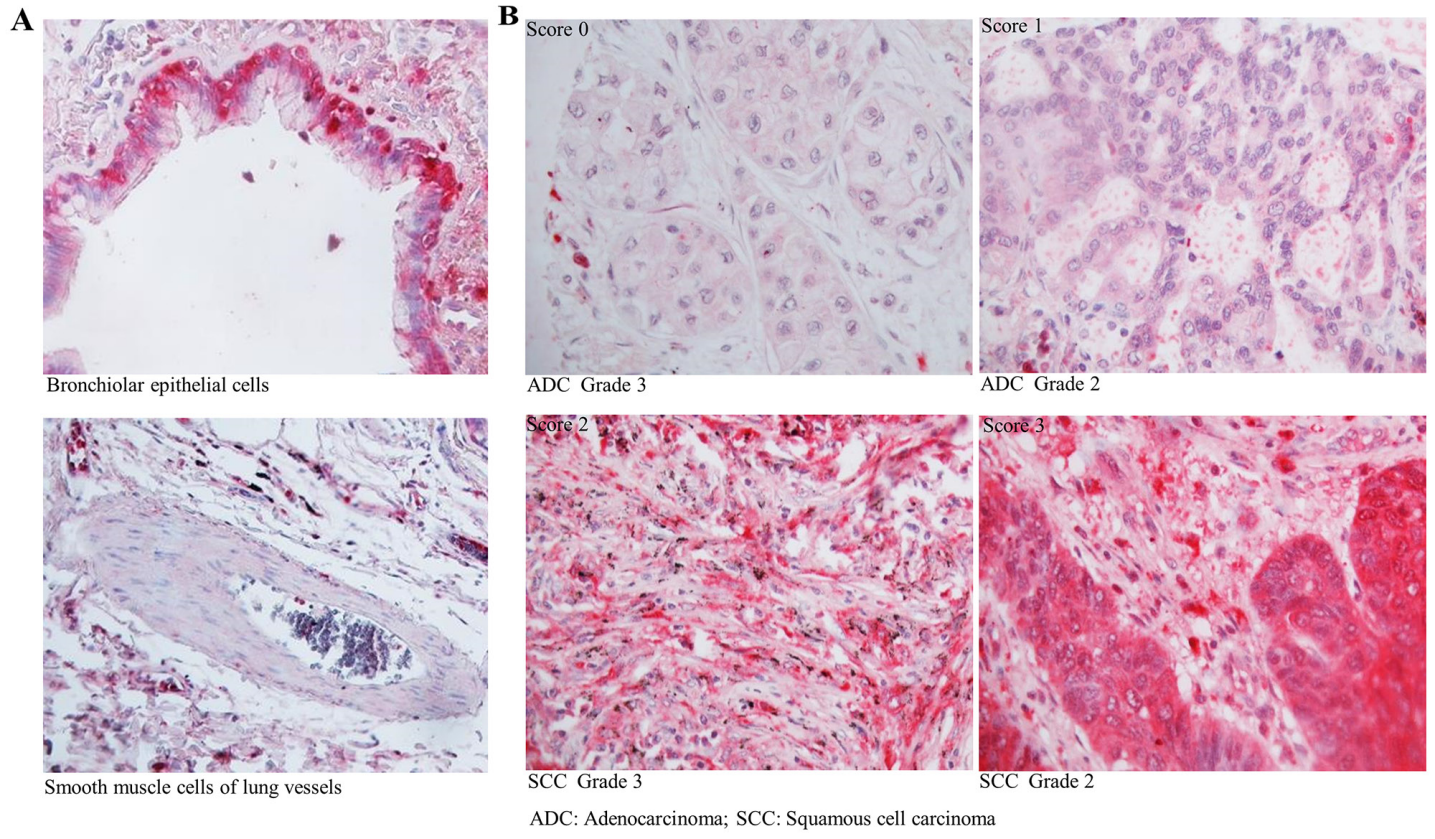
Expression of CSTA in normal lung tissue and primary lung tumor **(A)** Representative IHC results of CSTA protein expression (400 x magnification) in normal bronchiolar epithelial cells and smooth muscle cells of lung vessels. **(B)** Representative IHC results of CSTA protein expression (400 x magnification) in primary lung tumor.

Protein expression of CSTA was analyzed by immunohistochemistry (IHC) in normal human lung tissue and 228 lung tumor samples. Representative IHC results of CSTA protein expression in normal lung tissues and tumor tissues are shown in Figure 2. Strong CSTA staining was found in normal bronchiolar epithelial cells and was also detectable with lower intensity in alveolar macrophages and endothelial cells, while no expression of CSTA was found in smooth muscle cells of lung vessels. In lung tumors, positive staining of CSTA was found in 79.14% (110/139) of SCC and 37.68% (26/69) of ADC, and the difference was statistically significant (*p*<0.0001) (Table 1). Moreover, high expression of CSTA was inversely associated with tumor grade (*p<*0.01). We further analyzed the relationship between CSTA expression and tumor grade in SCC subtype and found that higher CSTA expression was significantly correlated with lower grade (*p*=0.031) (Table 2). However, Kaplan-Meier survival analysis did not reveal a significant impact of CSTA expression on survival (data not shown).

**Table 1:** Correlation between CSTA protein expression and clinicopathological parameters in primary lung tumor

		CSTA		p value
		Negative	Positive	
**Type**	SCC	29	110	
	ADC	43	26	**<0.00001**
	Others	11	9	
**Gender**	Male	69	125	0.531
	Female	14	20	
**pT**	1-2	68	115	0.717
	3-4	14	27	
**pN**	0	48	85	0.93
	1-3	33	57	
**Grade**	1-2	33	94	**0.001**
	3-4	39	42	

ADC, adenocarcinoma; SCC, squamous cell carcinoma.

**Table 2:** Correlation between CSTA protein expression and tumor grade in primary lung squamous cell carcinoma and adenocarcinoma

Type			CSTA		p value
			Negative	Positive	
**SCC**	Grade	1-2	15	80	**0.031**
		3-4	14	30	
**ADC**	Grade	1-2	18	14	0.333
		3-4	25	12	

SCC, squamous cell carcinoma; ADC, adenocarcinoma.

Taken together, CSTA is down-regulated in lung cancer cell lines, and CSTA has a potential diagnostic value in sub-classification of non-small cell lung cancer (NSCLC).

### Down-regulation of CSTA is correlated with DNA methylation and histone acetylation

**Figure 3:**
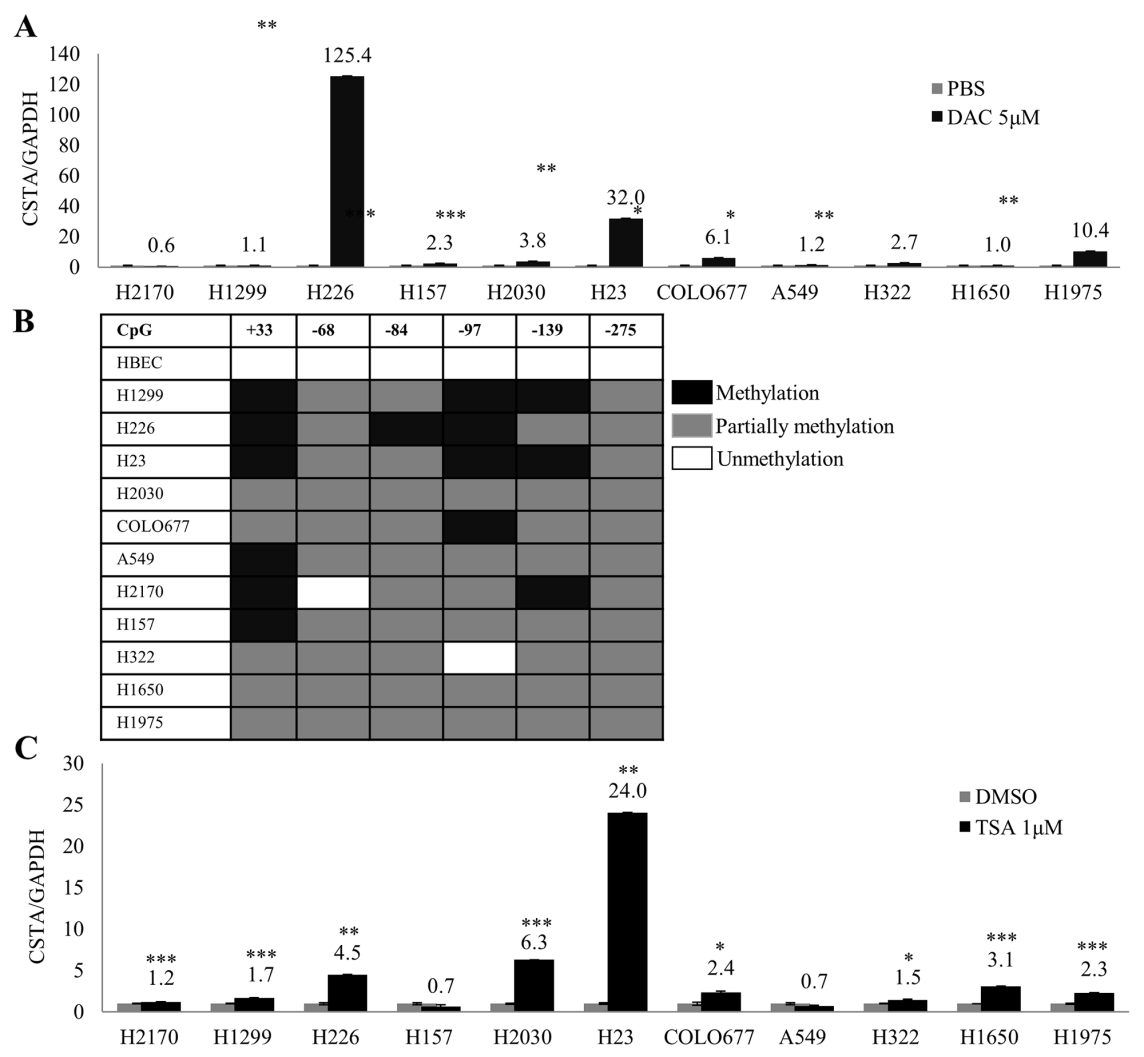
Epigenetic regulation of CSTA in lung cancer cells **(A)** mRNA expression of *CSTA* in 11 lung cancer cell lines was analyzed by real-time RT-PCR after treatment with 5 μM of DAC for 4 days. *CSTA* mRNA expression increased in most of the cell lines tested. The measurements are expressed as mean values of three independent experiments ± standard deviation. ^*^ p ≤ 0.05, ^**^ p ≤ 0.01, ^***^ p ≤ 0.001. **(B)** Methylation status of *CSTA* DNA in promoter and exon 1 regions determined by BS. **(C)** Real-time RT-PCR analysis after the cell lines were treated with 1 μM of TSA for 24 h. Up-regulation of *CSTA* mRNA was observed in almost all the cell lines with different levels. ^*^ p ≤ 0.05, ^**^ p ≤ 0.01, ^***^ p ≤ 0.001.

To investigate whether methylation and histone acetylation play a role in *CSTA* gene silencing, demethylation and deacetylation tests were performed. DNA (cytosine-5)-methyltransferase 1 (DNMT1) acts primarily as a maintenance methyltransferase by copying existing methylation patterns following DNA replication, the demethylation agent DAC is an analogue of cytosine that inhibits DNA methyltransferases including DNMT1 [21]. We found that DAC treatment led to a remarkably reduced DNMT1 protein expression, indicating a successful DAC moderation (Supplementary Figure 1B). Real-time RT-PCR showed that *CSTA* expression was extremely increased in five cell lines (H226, H23, H1975, COLO677 and H2030) and slightly up-regulated in H157, A549 and H322; whereas no CSTA re-expression was detected in H2170, H1299 and H1650 (Figure 3A). In COLO677, H23, H2030 and H226, we did not detect the restoration of CSTA on protein level (Supplementary Figure 1C) suggesting that DAC mediated CSTA re-expression was transcriptionally regulated. To further confirm the role that DNA methylation plays in *CSTA* gene expression, we knocked down DNMT1 (Supplementary Figure 2A, left) in 4 lung cancer cell lines, and found that down-regulation of DNMT1 led to a significantly increased CSTA expression in H23 and COLO677, but not H226. We did not observe any alteration after knockdown of DNMT1 in H2170 (Supplementary Figure 2A, right), which served as negative control, since DAC treatment did not cause enhanced *CSTA* mRNA expression in this cell line (Figure 3A).

To analyze detailed methylation status of CSTA, bisulfite sequencing (BS) was performed in eleven lung cancer cell lines. It turned out that *CSTA* DNA was partially methylated (only one allele was methylated) in the promotor and exon 1, while *CSTA* DNA was unmethylated in HBEC (Figure 3B).

After treatment with TSA, we found that CSTA expression was significantly up-regulated in six lung cancer cell lines (H23, H2030, H226, H1650, COLO677 and H1975) and slightly increased in three cell lines (H2170, H1299 and H322), whereas no CSTA restoration was detected in H157 and A549 (Figure 3C).

Our data suggest that epigenetic regulation including DNA methylation and histone acetylation are related to *CSTA* gene silencing, however, other mechanisms might also exist.

### CSTA overexpression results in a decreased activity of cathepsin B

**Figure 4:**
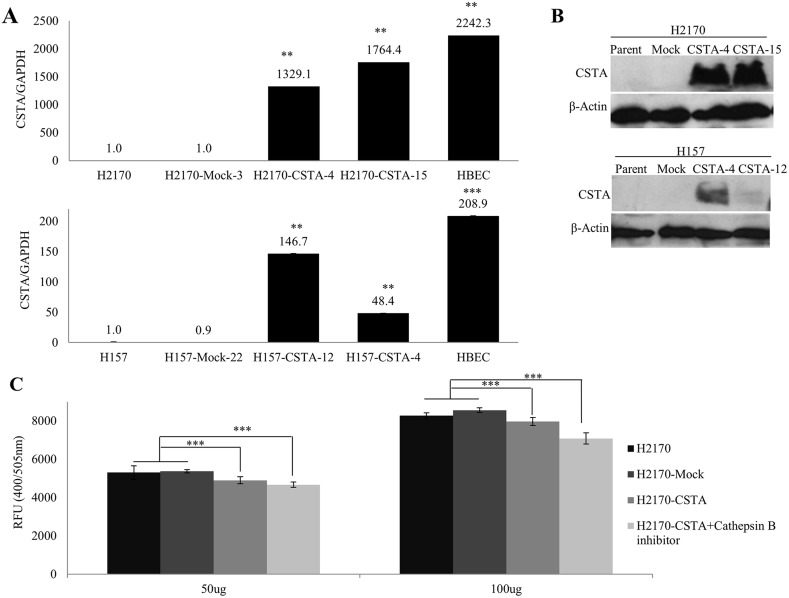
Overexpression of CSTA inhibits cathepsin B activity **(A)** *CSTA* mRNA expression after stable transfection was confirmed by real-time RT-PCR in H2170 and H157. ^**^ p ≤ 0.01, ^***^ p ≤ 0.001. **(B)** CSTA protein expression after stable transfection was confirmed by WB in H2170 and H157. **(C)** CSTA overexpression results in a decreased activity of cathepsin B in H2170. Error bar represent the means of three independent experiments. ^***^ p ≤ 0.001 when analyzed with Student's *t*-test.

After stable transfection with the CSTA expression vector, we detected CSTA mRNA and protein expression by real-time RT-PCR with 1329.1 and 1764.4 fold changes in H2170 transfectants as well as 146.7 and 48.4 fold changes in H157 transfectants. However, the overexpression of CSTA did not reach to the physiological level as shown in HBEC (Figure 4A). The overexpression of CSTA protein was confirmed by WB (Figure 4B).

The lysosomal cysteine protease cathepsin B has been considered an inhibitory target of CSTA and interacts with CSTA [22]. The results from real-time RT-PCR and WB revealed that cathepsin B was expressed in all eight lung cancer cell lines and HBEC. Compared to HBEC, cathepsin B was down-regulated on mRNA and protein levels (Supplementary Figure 2B). After stable transfection, we found that the activity of cathepsin B was markedly decreased in CSTA transfectant cells compared with mock transfectant cells (Figure 4C). However, this alteration was only detected in H2170 but not H157 (data not shown).

### CSTA inhibits cancer cell colony formation, migration and invasion through modulating the MAPK and AKT pathways

**Figure 5:**
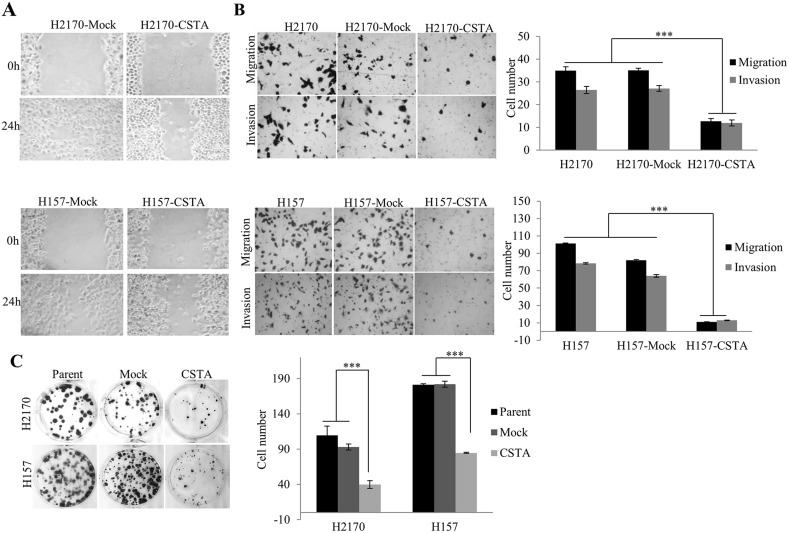
The effect of CSTA on proliferation, motility, migration and invasion in H2170 and H157 **(A)** CSTA overexpression prevents cell motility revealed by monolayer wound healing assay (50x objective). **(B)** Transwell migration and matrigel invasion assays showed that CSTA overexpression inhibited cell migration and invasion (50x objective). Right: Representative photos of migrated and invaded cells in H2170 and H157. Left: The migrated and invaded cells were quantified. The data presented are the means ± SE from three independent experiments. ^***^ p ≤ 0.001 when analyzed with Student's *t*-test. **(C)** CSTA overexpression inhibits the cell proliferation and viability by using colony formation assay. Right: Representative photos of colony formation assay in H2170 and H157. Left: Quantification of colony formation. ^***^ p ≤ 0.001.

To examine the effect of CSTA on cell motility, monolayer wound healing assay was carried out. The results showed that CSTA transfectant cells spread much more slowly than mock transfectant cells (Figure 5A).

Similar to wound healing assay, cell migration and invasion assay showed that the number of migrated and invaded tumor cells was significantly reduced in CSTA transfectant cells compared to control cells (Figure 5B).

Colony formation assay revealed that the number of colonies formed by the CSTA transfectant cells was significantly reduced compared with mock transfectant cells and parent cells (Figure 5C).

**Figure 6:**
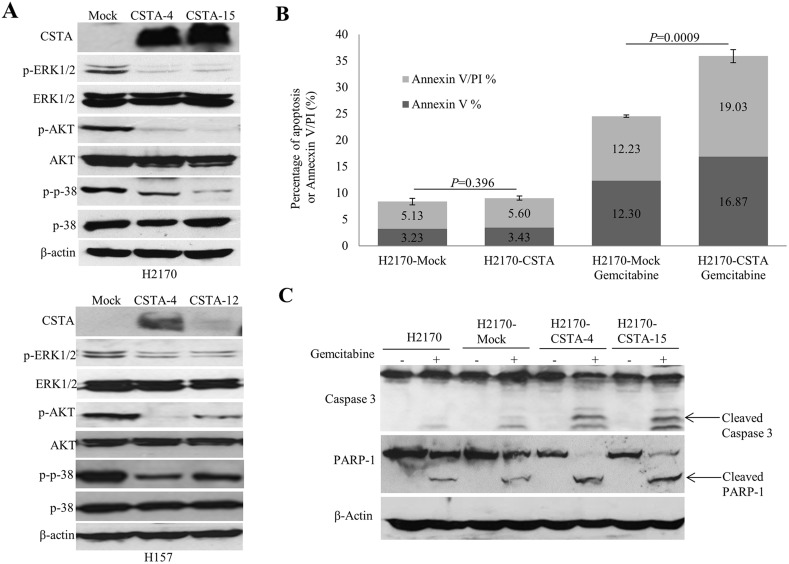
CSTA overexpression regulates PI3K/AKT and MAPK pathways and enhances gemcitabine-induced apoptosis **(A)** WB analysis showed that CSTA overexpression led to reduced phosphorylated levels of ERK and p38, two important elements of the MAPK pathway, as well as a decreased phosphorylated level of AKT in both H2170 and H157. **(B)** Quantitation of apoptotic cells represents the percentage of cells in each quadrant. Bar graphs depict the mean percentages of Annexin V positive apoptotic cells from three independent experiments. **(C)** Apoptotic markers (cleaved caspase 3 and cleaved PARP) were detected by WB.

To investigate the mechanism for CSTA-mediated cell growth inhibitory effects, we analyzed the mitogen-activated protein kinase (MAPK) and phosphoinositide-3-Kinase (PI3K) /AKT pathways. As shown in Figure 6A, CSTA overexpression led to a reduced phosphorylated AKT, phosphorylated ERK1/2 and phosphorylated p38 in both H2170 and H157. In addition, CSTA overexpression led to a reduced phosphorylated S6 (pS6) level in H157 but not in H2170 (Supplementary Figure 3).

Taken together, CSTA overexpression inhibits tumor cell growth, migration and invasion through modulating the MAPK and AKT pathways.

### CSTA enhances gemcitabine-induced apoptosis

To determine the influence of CSTA on gemcitabine-induced apoptosis, flow cytometry and WB were performed. Cells treated with apoptotic inducer H_2_O_2_ were used as positive control (data not shown). It turned out that the percentage of apoptotic cells was significantly increased in the CSTA transfectant cells (35.9%) compared to mock transfectant cells (24.53%) after gemcitabine treatment (*p*=0.0009), while there was no difference between the CSTA transfectant cells (8.36%) and mock transfectant cells (9.03%) without drug treatment (Figure 6B). The data suggest that CSTA overexpression enhanced gemcitabine-induced apoptosis in H2170.

To confirm this result, we analyzed cleaved caspase 3 and cleaved PARP1, two apoptotic markers, by WB. In line with the elevated apoptotic levels detected by flow cytometry in H2170, we found that, after gemcitabine treatment, the levels of cleaved caspase 3 and cleaved PARP1 were increased in CSTA transfectant cells compared with mock transfectant and parent cells (Figure 6C). However, the same phenomenon was not observed in H157 (data not shown).

### CSTA overexpression leads to an alteration in morphology and expression levels of EMT markers

**Figure 7:**
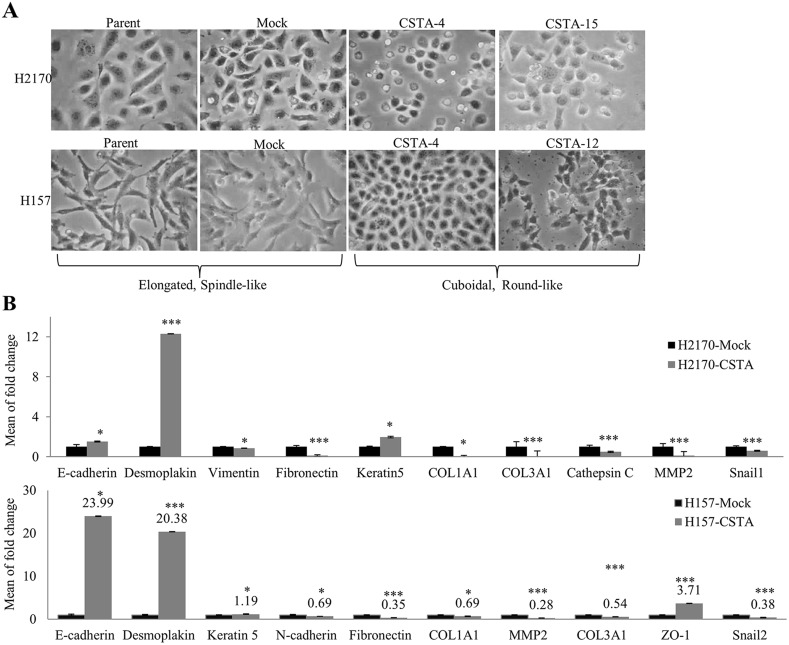
CSTA overexpression leads to an alteration in morphology and the expression of EMT markers **(A)** Ectopic CSTA expression resulted in morphological alteration. Mock transfectants and parent cells had a more elongated, spindle-like mesenchymal shape, while the CSTA transfectant cells looked rounded and had a cuboidal-like epithelial shape (100x objective). **(B)** The expression of EMT markers was determined by real-time RT-PCR in H2170 and H157. ^*^p ≤ 0.05, ^***^p ≤ 0.001 when analyzed with Student's *t*-test.

After transfection with CSTA, we observed a morphological alteration. Mock transfectant and parent cells had a more elongated, spindle-like mesenchymal shape, while the CSTA transfectant cells looked rounded and had a cuboidal-like epithelial shape (Figure 7A), indicating that cells are undergoing mesenchymal to epithelial transition (MET). CSTA overexpression thus seemed to induce a reversal of an EMT.

To further verify the hypothesis that CSTA overexpression might induce MET, we analyzed several EMT markers by real-time RT-PCR. It turned out that CSTA overexpression resulted in a decreased expression of mesenchymal markers (Fibronectin, Vimentin, Snail1, COL1A1, COL3A1, Cathepsin C and MMP2) and an increased expression of epithelial markers (Desmoplakin and Keratin 5) in H2170 (Figure 7B). In the CSTA stable transfected H157 cells, a decreased expression of mesenchymal markers (N-cadherin, Fibronectin, Snail2, COL1A1, COL3A1 and MMP2) and an increased expression of epithelial markers (E-cadherin, Desmoplakin, ZO-1 and Keratin 5) were found (Figure 7B).

**Figure 8:**
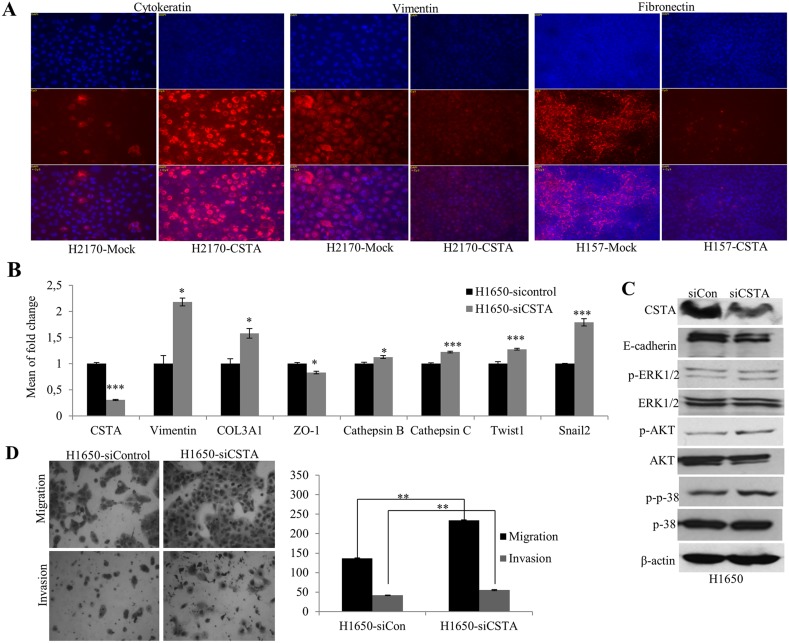
CSTA ectopic expression results in altered EMT markers on protein levels and CSTA knockdown leads to enhanced tumor cell migration and invasion as well as altered mRNA expression of EMT markers **(A)** Altered protein levels of EMT markers were detected by IF. An increased expression of the epithelial marker Cytokeratin and a decreased expression of the mesenchymal marker Vimentin were found in H2170 transfectant cells, while in H157 transfectant cells, a decreased expression of Fibronectin, a mesenchymal marker, was found. **(B)** Altered mRNA expression of EMT-related genes was detected by real-time RT-PCR after CSTA knockdown in H1650. **(C)** CSTA knockdown leads to increased activity of AKT and MAPK pathways and a reduced protein expression of the epithelial marker E-cadherin in H1650. **(D)** CSTA knockdown promotes the cell migration and invasion. Left: Representative images of migrated and invaded cells (50x objective); Right: Quantification of migrated and invaded cells. ^**^ p ≤ 0.01 when analyzed with Student's *t*-test.

We further performed immunofluorescence (IF) to confirm the protein expression of EMT markers. A prominently increased expression in Cytokeratin and a decreased expression in Vimentin were observed in H2170 (Figure 8A). In H157, a significantly decreased Fibronectin was found in CSTA transfectant cells compared with mock cells (Figure 8A).

Collectively, CSTA overexpression prevents the process of EMT in lung cancer cells.

### CSTA knockdown promotes cell migration, invasion and EMT

To analyze whether down-regulation of CSTA could lead to a reversed effects on cell migration and invasion, we knocked down CSTA in H1650 by siRNA. A successful siRNA knockdown was confirmed with a dramatic decrease of CSTA on both mRNA and protein levels (Figure 8B and 8C) and correspondingly, the number of migrated and invaded cells was significantly increased in CSTA knockdown cells compared with control (Figure 8D).

To explore the effect of CSTA knockdown on EMT, again, the expression of EMT markers was analyzed. As shown in Figure 8B, CSTA knockdown resulted in an increased expression of mesenchymal markers (Vimentin, Twist1, Snail2 and COL1A1) and a decreased expression of epithelial markers (E-cadherin and ZO-1). Meanwhile, cathepsin B and C were also increased.

Moreover, we found that CSTA down-regulation led to an enhanced level of phosphorylated p38, a major player of the MAPK pathway, and an increased expression of phosphorylated AKT (Figure 8C).

Taken together, our data suggest that CSTA acts as a tumor suppressor and inhibits tumor cell growth through inhibiting EMT in lung cancer cells.

### CSTA overexpression prevents TGF-β1 induced EMT through inhibiting ERK/MAPK pathway

**Figure 9:**
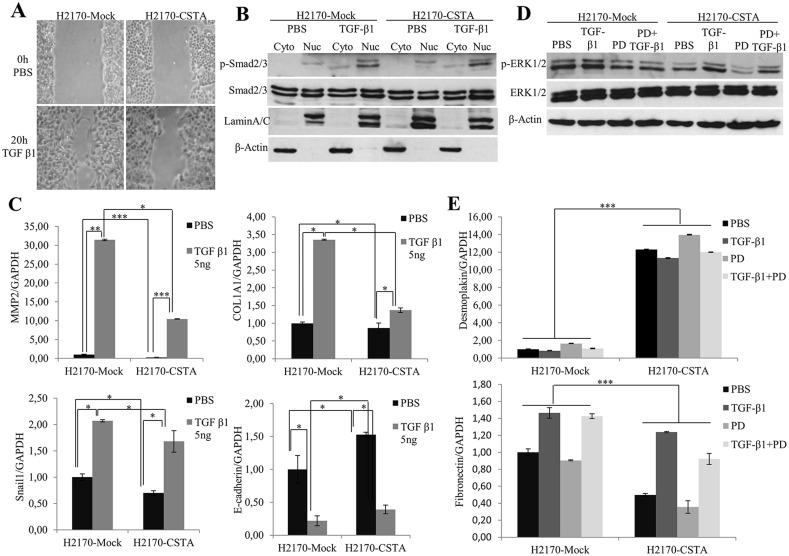
CSTA overexpression prevents TGF-β1 induced EMT through inhibiting ERK/MAPK pathway **(A)** After TGF-β1 treatment, CSTA transfectant cells showed a reduced motility compared to mock cells as revealed by monolayer wound healing assay. **(B)** Enhanced phosphorylation level of SMAD2/3 was detected by WB analysis in both CSTA transfectant cells and mock cells after TGF-β1 treatment. LaminA/C was used as a nuclear loading control and β-Actin was used as a cytoplasmic loading control. **(C)** The mRNA expression of EMT markers after TGF-β1 induction. Real-time RT-PCR showed that TGF-β1 induction led to a decreased level of the epithelial marker E-cadherin and increased levels of mesenchymal markers like Snail1, MMP2 and COL1A1, which could be prevented by overexpression of CSTA. ^*^ p ≤ 0.05, ^**^ p ≤ 0.01, ^***^ p ≤ 0.001. **(D)** ERK/MAPK pathway was determined by WB after treatment with TGF-β1, PD98059, or in combination of both. TGF-β1 treatment resulted in an increased phosphorylation of ERK1/2, which could be inhibited by MEK inhibitor PD98059 in both CSTA transfectant cells and mock cells. However, compared to mock cells, CSTA transfectants exhibited reduced phosphorylation level of ERK1/2. **(E)** Analysis of EMT markers by real-time RT-PCR after treatment with TGF-β1, PD98059, or in combination of both. ^***^ p ≤ 0.001.

TGF-β signaling is associated with EMT in various cancers including NSCLC [23]. To examine the effect of CSTA overexpression on TGF-β1-induced EMT, wound healing assay was performed. Figure 9A shows that the CSTA transfectant cells spread along the wound edges much more slowly than control cells after treatment with TGF-β1.

TGF-β induces EMT through canonical SMAD-dependent and SMAD-independent signaling [24]. In our study, we found an elevation of phosphorylated SMAD2/3 (Figure 9B). In line with this, increased mRNA levels of mesenchymal markers (Fibronectin, Snail, MMP2 and COL1A1) and a decreased expression of epithelial markers (Desmoplakin and E-cadherin) were observed (Figure 9C) after TGF-β1 treatment, indicating the activation of the TGF-β/SMAD/EMT pathway. However, when we compared the SMAD2/3 activity of CSTA transfectants to mock cells, we did not find any alteration (Figure 9B), instead, we found a reduced phosphorylation level of ERK1/2, a component of MAPK pathway (Figure 9D), suggesting that CSTA could play a role in activation of the TGF-β/MAPK/EMT pathway.

To confirm the involvement of MAPK pathway in the prevention of TGF-β induced EMT by CSTA, cells were treated with TGF-β1 and/or PD98059 (a mitogen activated protein kinase inhibitor). As shown in Figure 9D, the increased level of phosphorylated ERK1/2 by TGF-β1 induction could be blocked by PD98059 in both CSTA and mock transfectant cells, and compared to mock cells, CSTA transfectants exhibited lower p-ERK1/2 level. Moreover, an increased expression of the epithelial marker Desmoplakin and a decreased expression of the mesenchymal marker Fibronectin were observed in CSTA transfectant cells after PD98059 treatment (Figure 9E).

Taken together, the data indicate that TGF-β induced EMT could be prevented by CSTA-mediated inactivation of the MAPK pathway in lung cancer cells.

## DISCUSSION

Initial studies on cystatins focused on their role as inhibitors of cysteine proteases [5]. In addition, loss-of-function mutations in CSTA were identified as cause of exfoliative ichthyosis, highlighting the expression of CSTA in keratinocytes and a role in desmosome-mediated cell-cell adhesion [6]. Interestingly, both increased cysteine protease inhibition and cystatin A expression were associated with better survival in NSCLC [25], and enforced expression of CSTA reduced metastasis in breast cancer [26]. In this study, we performed a comprehensive analysis on the role of CSTA in lung cancer.

Firstly, we found that down-regulation of CSTA was a frequent molecular event in lung cancer cells. In line with previous report [25], primary SCC expressed significantly higher levels of CSTA compared to ADC suggesting that it could be a valuable biomarker for lung SCC. This can be explained by the observations that the gene is expressed in squamous cells [6] and it is located on 3q21.1, a chromosomal region that is typically gained in lung SCC but not ADC [27–29]. In accordance with the loss of CSTA in all of the SCC cell lines that we investigated, we found that CSTA was lowly expressed in lung SCC with higher grade, reflecting the fact that cancer cell lines are usually derived from primary tumors with poor differentiation and/or advanced stage. In contrast, Werle *et al*. reported a counterintuitive positive correlation between CSTA expression and tumor grade [25]. The associations between grading, survival and CSTA expression deserves further studies.

Secondly, we investigated the mechanisms responsible for CSTA silencing. Actually CSTA lacks typical CpG islands in the promoter region [30]. However, several lines of evidence show that genes without CpG islands could also be regulated by epigenetic mechanism [30, 31]. In our study, demethylation test, deacetylation test together with BS clearly revealed that CSTA silencing could be at least partially attributed to DNA methylation and histone acetylation. Nevertheless, other mechanisms might also be included. To our knowledge, this is the first report on the epigenetic regulation of CSTA in human cancer.

Thirdly, the function of CSTA was explored. So far few publications indicate the tumor suppressive function of CSTA in esophageal cancer [32]. CSTA is an inhibitor of cathepsins involved in the ECM remodeling through degradation of ECM components [33]. We selected the two lung squamous carcinoma cell lines H2170 and H157 for stable transfection based on the reasons 1) no expression of CSTA was found in these two cell lines, 2) the transfection conditions were well established in these two cell lines, 3) they are tumorigenic as revealed by nude mice test (data not shown). We found that re-expression of CSTA in H2170 resulted in decreased activity of cathepsin B compared with mock cells. In addition, re-expression of CSTA had an inhibitory effect on cell growth. Inversely, knockdown of CSTA up-regulated mRNA expression of cathepsin B and promoted cell migration and invasion. These data point to the role of CSTA as a tumor suppressor in lung cancer cells.

Also the increased sensitivity of lung cancer cells after CSTA transfection to gemcitabine-induced apoptosis is compatible with a tumor suppressive function. Previous studies showed that CSTA suppressed ultraviolet B- and salt-induced cell death by apoptosis [34, 35]. Gemcitabine, one of the chemotherapeutic agents, suppresses the growth of cancer cell through induction of apoptosis [36]. We found that ectopic expression of CSTA resulted in an enhanced apoptotic activity after gemcitabine treatment. We suppose, cellular context might be essential to explain different roles that CSTA plays in apoptosis.

Fourthly, we observed a morphological change in cells from mesenchymal-like pattern to epithelial-like pattern upon CSTA overexpression by stable transfection. This phenomenon was accompanied by molecular alterations of EMT markers with upregulated epithelial markers and down-regulated mesenchymal markers. On the contrary, knockdown of CSTA led to the inverse results, indicating an important role of CSTA in MET. Indeed, the inhibitory effects of CSTA in tumor motility are in good agreement with its role in MET, since EMT is believed to be associated with tumor invasion and metastasis. In line with this, other cystatin family members including cystatin C, cystatin D and cystatin SN were found to be involved in EMT [17–19]. Cystatin C seems to act on EMT via its binding affinity to the TGF-β receptor II and a pathophysiological role independent of its protease inhibitory function is well established in cystatin C [37–39].

Finally, we investigated the molecular mechanisms underlying CSTA-mediated cell growth inhibitory effects. We found that CSTA suppresses cell growth through inhibiting the MAPK and AKT pathways. In addition, TGF-β signaling is a key effector of EMT [40]. It was shown that cystatin C suppresses TGF-β induced EMT in a SMAD-dependent manner [17]. We investigated the influence of CSTA overexpression on TGF-β1-induced EMT and found that ectopic expression of CSTA led to a decreased activity of ERK1/2, which could be inhibited by MEK inhibitor PD98059, while the activity of SMAD2/3 did not change. Correspondingly we observed the alteration of EMT markers after TGF-β1 and PD98059 modification in comparison of CSTA transfectants with mock cells. Based on these findings, we speculate that CSTA prevents TGF-β1-induced EMT by modulating the MAPK pathway in lung cancer cells. Interestingly, similar pathways and molecular targets are affected by cystatin C suggesting some analogy between different cystatins in EMT [39, 41].

The study indicates that CSTA harbors different functions being mediated not only through inhibition of lysosomal cysteine proteases but also other mechanisms. CSTA along with other cystatins merits further investigation based on their impact on various aspects of tumor biology and their potential in cancer treatment.

## MATERIALS AND METHODS

### Cell lines, cell culture and drug treatment

HBEC were obtained from Clonetics (San Diego, CA, USA) and cultured in BEG media (Lonza, Walkersville, USA). Human lung carcinoma cell lines, including small-cell lung cancer cell (COLO677, COLO668, H82, DMS79, H526, SHP77 and H123) and NSCLC cell lines (ADC: H1299, H2030, H23, A549, H322, H1650, H1975, D51, D54, H125, D117, D97 and BEN/97 as well as SCC: H2170, H226, H157), were purchased from the American Type Culture Collection (ATCC, Rockville, USA) or established in our laboratory (D51, D54, D117 and D97) [8]. Cells were grown in RPMI 1640 medium (Biochrom AG, Berlin, Germany) or Leibovitz 15 media (Thermo Fisher Scientific, Waltham, MA, USA) supplemented with 10% fetal bovine serum (Biochrom AG) and maintained in a humidified atmosphere with 5% CO2 at 37°C.

For demethylation, 5 μM of DAC (Sigma Chemical Co., St Louis, MO USA) was added into the medium on days 0, 2 and harvested in days 4 when cells around 60 % confluence. For deacetylation tests, cells were treated with 1 μM of TSA (Sigma) for 1 day.

To study the effect of TGF-β1 on EMT, cells were starved in serum-free medium overnight and treated with 5 ng/ml of human recombinant TGF-β1 (PeproTech, New Jersey, USA) and/or 10 μM of PD98059 (LC laboratories, Woburn, MA USA) for 24 h.

### RNA extraction and real-time RT-PCR

Total RNA was extracted from cells by using Trizol reagent (peqGOLD TriFastTM, Erlangen, Germany) according to the manufacturer's instruction. RNA reverse transcription and real-time RT-PCR were performed as described previously [42].

### Northern blot (NB)

NB analysis was performed as previously described [8].

### Signaling modification and bisulfite sequencing (BS)

Bisulfite modification and BS were carried out as described previously [43]. Bisulfite PCR primer was shown in Supplementary Table 1.

### Protein extraction and western blot (WB)

Cytoplasmic, nuclear or total proteins were extracted by using a Nuclear Extract Kit (Active Motif, Rixensart, Belgium) and the concentration was determined by applying a BCA Protein Assay kit (Thermo Fisher Scientific) according to the manufacturer's protocol. WB was performed as described previously [44]. Information about primary and secondary antibodies is shown in Supplementary Table 2.

### Tissue microarray (TMAs) construction and immunohistochemistry (IHC)

In total 228 of lung tumors samples were constructed for TMAs as described previously [43]. The study was approved by the local ethic committee (Nr.3815-07/13).

Three μm sections were cut from the TMA blocks, placed on glass slides and were dewaxed with xylene and gradually hydrated. After antigen retrieval, the slide was blocked by using the Biotin Blocking System (Agilent Technologies, Santa Clara, CA USA), then incubated with primary antibody (Supplementary Table 2) overnight. Detection was performed based on the protocol of Dako REAL Detection System (Agilent Technologies). IHC was scored semi-quantitatively as negative (<10% positively stained cells; score 0), weak (10–25% positively stained cells; score 1), moderate (26–50% positively stained cells; score 2) or strong (more than 50% positively stained cells; score 3). For the statistical analysis, scores 0 was interpreted as negative staining, while scores 1, 2 and 3 together were considered positive.

### CSTA expression vector construction and stable transfection

The full-length cDNA of CSTA (NM_005213) was amplified by PCR using primers listed in Supplementary Table 1 including the restriction enzyme cutting sites HindIII and BamHI. The PCR product and empty vector pcDNA3.1HisB (Invitrogen, Carlsbad, USA) were respectively digested with HindIII and BamHI (Promega, Madison, USA) and then ligated by T4 DNA ligase (Biolabs, New England, UK). The insert sequence and orientation were confirmed by sequencing.

H2170 and H157 without endogenous expression of CSTA were transfected with pcDNA3.1HisB/CSTA expression vector using transfection reagent lipofectamine 2000 (Invitrogen) according to the manufacturer's suggestions. Stable transfectants were picked out by the addition of G418 (0.4 mg/ml) for 2-3 weeks.

### RNA interference and transient transfection

H1650 with endogenous expression of CSTA was transfected with human CSTA siRNA (sc-44430, Santa Cruz, CA, USA) and control siRNA (sc-37007, Santa Cruz) using lipofectamine 2000 (Thermo Fisher Scientific) for 48 h.

### Cathepsin B activity assay

Cathepsin B activity assay was performed by using a Cathepsin B Activity Assay Kit (Promokine, Heidelberg, Germany) according to the manufacturer's protocol and the activity was measured using a fluorometer plate reader (TECAN, infinite, M1000PRO).

### Colony formation, wound-healing, migration and invasion assay

Colony formation, wound-healing, migration and invasion assays were performed as previously described [42, 45].

### Annexin V-FITC apoptosis analysis

Apoptotic assay was carried out by applying an Annexin V-FITC apoptosis detection kit (Abcam, Cambridge, UK) according to the manufacturer's instruction. Cells were incubated with apoptosis inducer, gemcitabine (100 μM for H2170; 500 μM for H157), for 48 h. Cells treated with H_2_O_2_ (10mM; for 3 h) were used as positive control. Flow cytometry analysis was performed by using a FACSCanto II flow cytometer (BD Biosciences, Franklin Lanes, NJ, USA).

### Immunofluorescence (IF)

Cells were grown on 8-well chamber slides. After reaching 90% confluence, slides were fixed with −20°C acetone/methanol for 2 min. After air drying, slides were immediately rinsing in TBS. Primary antibodies (Supplementary Table 2) diluted in in Antibody Diluent/Background Reducing solution (Agilent Technologies) were applied and incubated overnight at 4°C. For detection of bound antibodies, incubation with a Cy3 labelled goat anti mouse polyclonal antiserum (Dianova GmbH, Germany) was performed. Coverslips were mounted using Mounting Medium containing DAPI (Vector Laboratories, Burlingame, CA USA). Samples were analyzed and photographed using an Axiophot microscope and the AxioVision software (Carl Zeiss MicroImaging GmbH, Jena, Germany).

### Statistical analysis

The correlation between CSTA expression and clinicopathological parameters was assessed by the two-tailed chi-square or Fisher's exact test using the software package SPSS 21.0 (SPSS, Chicago, USA). Student's *t*-test was carried out to detect the difference between CSTA transfectant and mock tansfectant cells. Overall survival was analyzed using Kaplan-Meier curves with log-rank test. P values less than 0.05 were regarded as statistically significant.

### Ethics approval and consent to participate

The study was approved by the ethic committee of University Hospital Jena Germany (3815-07/13).

## SUPPLEMENTARY MATERIALS FIGURES AND TABLES



## Abbreviations

CSTA: cystatin
HBEC: human bronchial epithelial cells
DAC: 5-aza-2-deoxycytidine
TSA: Trichostatin A
BS: bisulfite sequencing
SCC: squamous cell carcinoma
ADC: adenocarcinoma
NSCLC: non-small cell lung cancer
MET: mesenchymal to epithelial transition
EMT: epithelial to mesenchymal transition
WB: western blot
TMA: tissue microarray
IHC: immunohistochemistry
IH: immunofluorescence
DNMT1: DNA (cytosine-5)-methyltransferase 1
MAPK: mitogen-activated protein kinase
PI3K: phosphoinositide-3-Kinase
S6: ribosomal protein S6 kinase
