# Supplementary material for: Cystatin A suppresses tumor cell growth through inhibiting epithelial to mesenchymal transition in human lung cancer
Source: Oncotarget. 2017 Dec 20;9(18):14084–98. doi: 10.18632/oncotarget.23505 (PMC5865655; doi:10.18632/oncotarget.23505)
Supplement: Supplementary file 1 [file oncotarget-09-14084-s001.pdf]

Cystatin A suppresses tumor cell growth through inhibiting epithelial to mesenchymal transition in human lung cancer

SUPPLEMENTARY MATERIALS

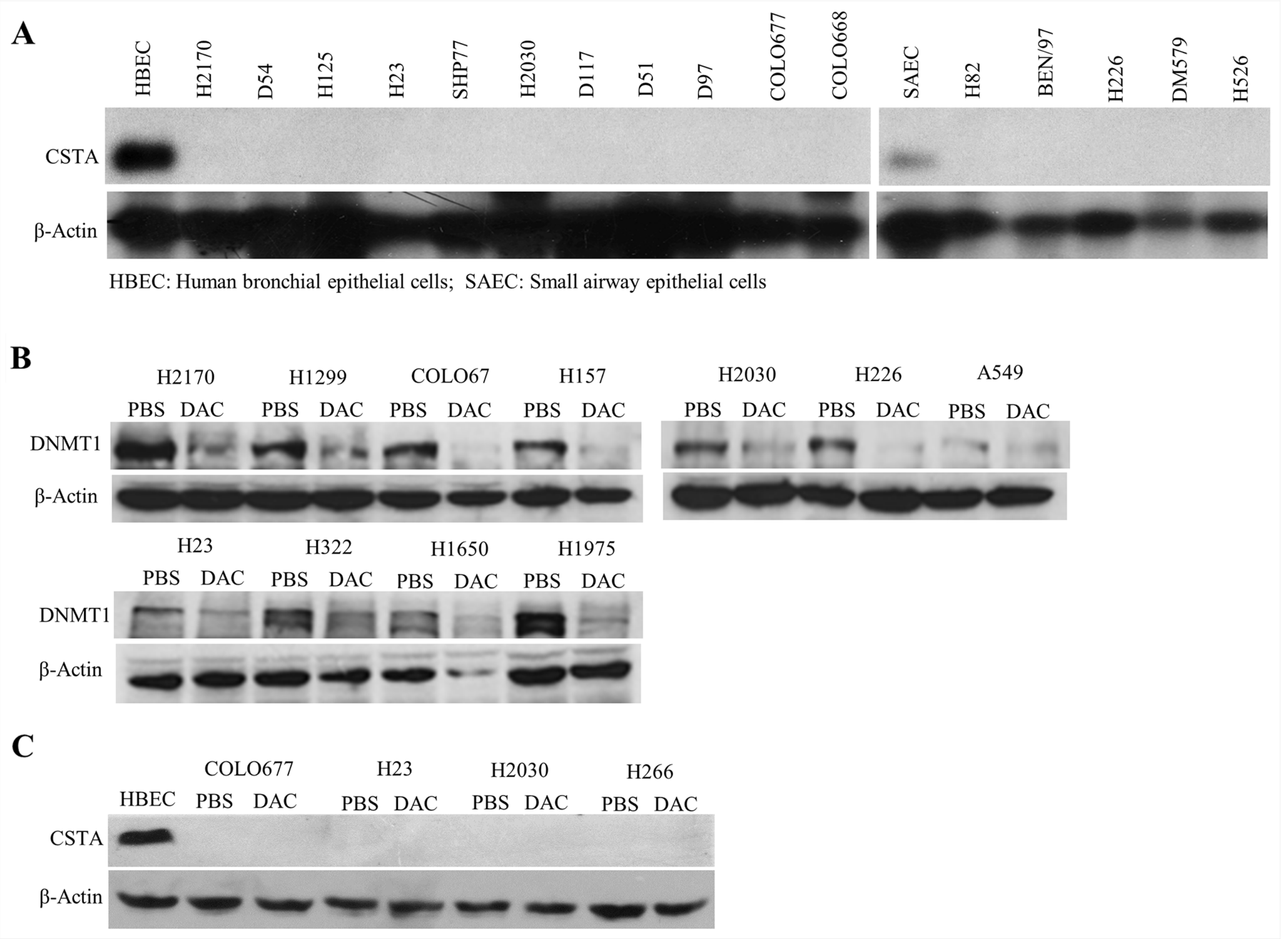

Supplementary Figure 1: (A) mRNA expression of *CSTA* in 16 lung cancer cell lines, 2 normal lung epithelial cells (HBEC and SAEC) was analyzed by northern blot. **(B)** DNMT1 expression was detected in lung cancer cell lines by WB after treatment with 5 µM of DAC for 4 days. **(C)** CSTA protein expression was analyzed in 4 lung cancer cell lines by WB after treatment with 5 µM of DAC for 4 days.

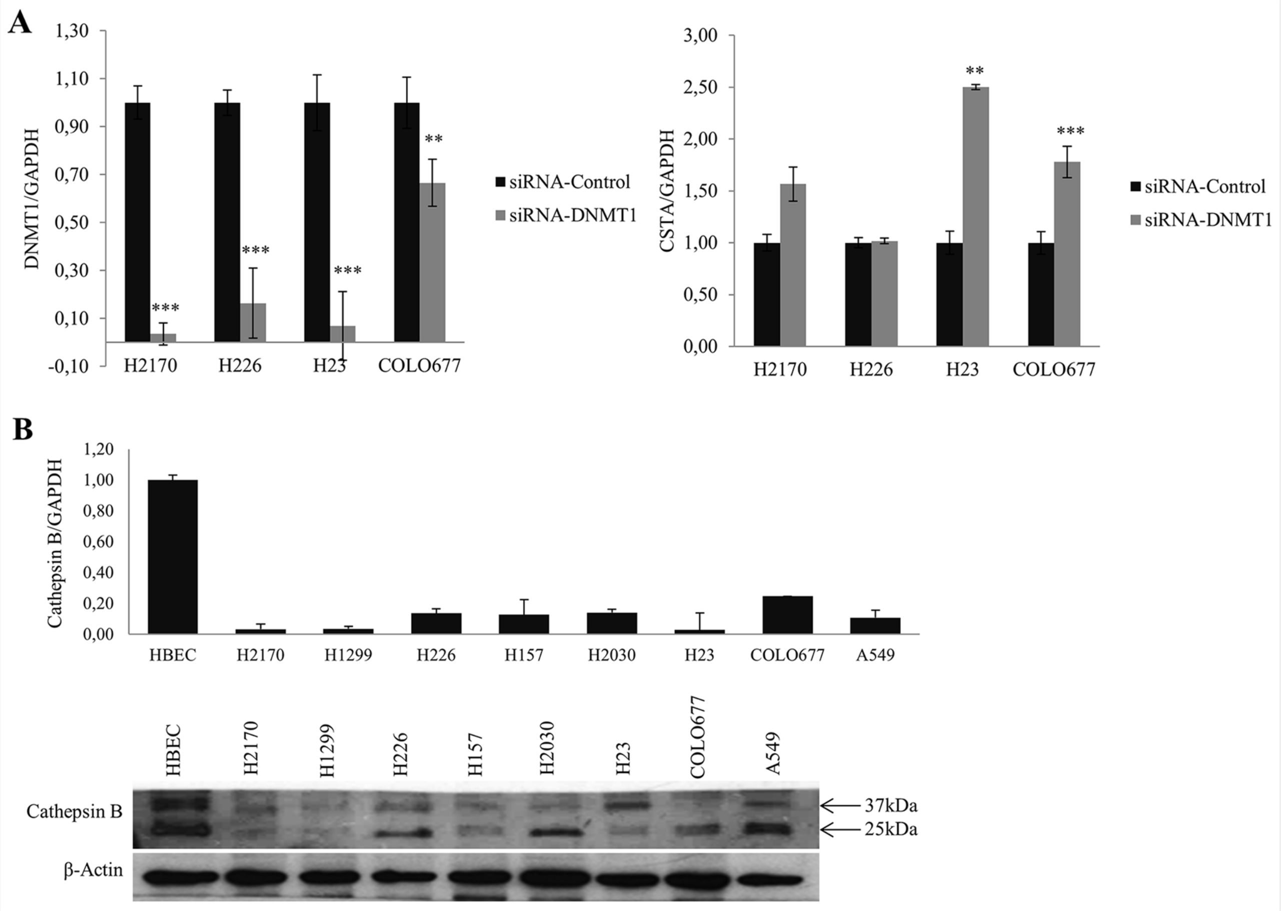

Supplementary Figure 2: **(A)** DNMT1 and CSTA expression was analyzed in 4 lung cancer cell lines by real-time RT-PCR after siRNA-DNMT1 knockdown. ** $p \leq 0.01$, *** $p \leq 0.001$. **(B)** mRNA and protein expression of cathepsin B in 8 lung cancer cell lines and normal cells (HBEC) were analyzed by real-time RT-PCR and WB. Two fragments representing (25 and 37 kDa) mature cathepsin B were detected.

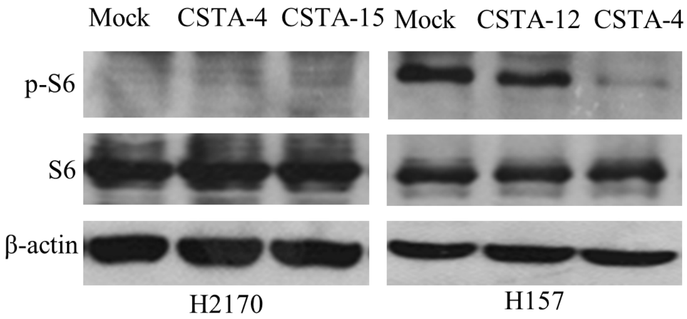

Supplementary Figure 3: S6 and phosphorylated S6 (pS6) were analyzed by WB in H157 and H2170.

Supplementary Table 1: Sequences of primers

PCR	Primer name	Sequence (5’-3’)
BS primer in promoter and exon regions		
	BS-F	5′-TAGATGATGTAATAGGATGG-3′
	BS-R	5′-CCTAAATAACATCAACTCAC-3′
PCR		
	CSTA-ORF-F	5′-CCCAAGCTTGGGATCGCCATGATACCTGGAGG-3′
	CSTA-ORF-R	5′-CGGGATCCCGTTAAACCTTATCGTCGTCATC-3′
Real-time RT-PCR		
	CSTA-F	5′-CGGAAAATTGGAAGCTGTGC-3′
	CSTA-R	5′-CAAGTCCTCATTTTGTCCGGG-3′
	GAPDH-F	5′-GACAGTCAGCCGCATCTTCT-3′
	GAPDH-R	5′-TTAAAAGCAGCCCTGGTGAC-3′
	E-cadherin-F	5′-TTCCCAACTCCTCTCCTG-3′
	E-cadherin-R	5′-AAACCTTGCCTTCTTTGTC-3′
	Desmoplakin-F	5′-GCTTGCCAACTTCAGAGGTTCT-3′
	Desmoplakin-R	5′-TTGGAGAATAGCCTGGAGCAGT-3′
	Keratin 5-F	5′-GCTGAGAAACATGCAGGACC-3′
	Keratin 5-R	5′-TGAACAAGGTGGAGCTGGAG-3′
	ZO-1-F	5′-CCTTACTCACCACAAGCGCA-3′
	ZO-1-R	5′-GCGAAAGGTAAGGGACTGGA-3′
	Vimentin-F	5′-GAAATTGCAGGAGGAGATGC-3′
	Vimentin-R	5′-GCAAAGATTCCACTTTGCGT-3′
	N-cadherin-F	5′-ATTGGACCATCACTCGGCTTA-3′
	N-cadherin-R	5′-CACACTGGCAAACCTTCACG-3′
	Fibronectin-F	5′-CCATAAAGGGCAACCAAGAG-3′
	Fibronectin-R	5′-AAACCAATTCTTGGAGCAGG-3′
	CLO1A1-F	5′-GTTGTGCGATGACGTGATCTGTGA-3′
	CLO1A1-R	5′-TTCTTGGTCGGTGGGTGACTCTG-3′
	COL3A1-F	5′-CCAGGAGCTAACGGTCTCAG-3′
	COL3A1-R	5′-CAGGGTTTCCATCTCTTCCA-3′
	MMP2-F	5′-ACCACAGCCAACTACGATGA-3′
	MMP2-R	5′-GTAAATGGGTGCCATCAGGG-3′
	Cathepsin B-F	5′-GTGGACATGAGCTACTTGAAG-3′
	Cathepsin B-R	5′-TCTCTTTGATGGTGGGACACT-3′
	Cathepsin C-F	5′-CCGGAAAGAAGGTGGGAACT-3′
	Cathepsin C-R	5′-TGCAGTCCAAGACTTCTGAAT-3′
	Snail1-F	5′-CGAGCTGCAGGACTCTAATCC-3′
	Snail1-R	5′-GTCCCAGATGAGCATTGGCAG-3′
	Snail2-F	5′-GCGAACTGGACACACATACAG-3′
	Snail2-R	5′-GCAGCGGTAGTCCACACAGT-3′
	Twist1-F	5′-CACGAGCGGCTCAGCTACG-3′
	Twist1-R	5′-CAGAGTCTCTAGACTGTCCAT-3′

BS, bisulfite sequencing.

Supplementary Table 2: Antibodies used for western blot (WB), immunohistochemistry (IHC) and immunofluorescence (IF)

Antibody	Host	Company	Dilution
Cystatin A (B-11):sc-376759	Mouse	Santa Cruz Biotechnology, Santa Cruz, CA USA	1:1000 (WB)
			1:750 (ICH)
β-actin: MAB1501R	Mouse	Milipore, Billerica, MA USA	1:1000 (WB)
Caspase-3: 9662	Rabbit	Cell Signaling Technology, Danvers, MA USA	1:1000 (WB)
PARP-1 (F-2):sc-8007	Rabbit	Santa Cruz Biotechnology, Santa Cruz, CA USA	1:1000 (WB)
Caspase-3 control cell extracts: 9663s		Cell Signaling Technology, Danvers, MA USA	
E-cadherin: clone 4A2C7	Mouse	Invitrogen GmbH, Karlsruhe, Germany	1:1000 (WB)
EDA^{+}-Fibronectin: sc-59826 (clone IST9)	Mouse	Santa Cruz Biotechnology, Santa Cruz, CA USA	1:500 (IF)
Cytokeratin: clone MNF116*	Mouse	Agilent Technologies	1:300 (IF)
Vimentin: clone V9	Mouse	Agilent Technologies	1:20 (IF)
Phospho-AKT: 9275s	Rabbit	Cell Signaling Technology, Danvers, MA USA	1:1000 (WB)
Phospho -p44/42 MAPK (ERK1/2): 9101s	Rabbit	Cell Signaling Technology, Danvers, MA USA	1:1000 (WB)
Phospho -p38 (Thr180/Tyr182): 4511s	Rabbit	Cell Signaling Technology, Danvers, MA USA	1:1000 (WB)
Phospho -S6 (Ser235/236): 4858s	Rabbit	Cell Signaling Technology, Danvers, MA USA	1:1000 (WB)
Phospho -Smad2 (Ser465/467)/ Smad3(Ser423/425):8828s	Rabbit	Cell Signaling Technology, Danvers, MA USA	1:1000 (WB)
AKT: 9297s	Rabbit	Cell Signaling Technology, Danvers, MA USA	1:1000 (WB)
p44/42 MAPK (Erk1/2): 4695s	Rabbit	Cell Signaling Technology, Danvers, MA USA	1:1000 (WB)
p38: 9212s	Rabbit	Cell Signaling Technology, Danvers, MA USA	1:1000 (WB)
Cycline D1 (H-295): sc-753	Rabbit	Santa Cruz Biotechnology, Santa Cruz, CA USA	1:1000 (WB)
Cathepsin B (S-12): sc-6493	Goat	Santa Cruz Biotechnology, Santa Cruz, CA USA	1:500 (WB)
S6 Ribosomal protein: 2217s	Rabbit	Cell Signaling Technology, Danvers, MA USA	1:1000 (WB)
Smad2/3:8685s	Rabbit	Cell Signaling Technology, Danvers, MA USA	1:1000 (WB)
DNMT1 (1-110): H00001786-M01	Mouse	Abnove GmbH, Heidelberg, Germany	1:500 (WB)
Peroxidase-conjugated anti-goat IgG: sc-2020	Rabbit	Santa Cruz Biotechnology, Santa Cruz, CA USA	1:1000 (WB)
Peroxidase-conjugated anti-mouse IgG	Rabbit	DAKO, Hamburg, Germany	1:1000 (WB)
Peroxidase-conjugated anti-rabbit IgG: sc-2030	Goat	Santa Cruz Biotechnology, Santa Cruz, CA USA	1:1000 (WB)

* Cytokeratin 5, 6, 8, 17 and 19.
